# RETRACTION: Effect of angiogenesis inhibitors on wound healing in patients with ovarian cancer: A meta‐analysis

**DOI:** 10.1111/iwj.70114

**Published:** 2024-10-17

## Abstract

Retraction: LiX. , ZengD. , and ShiJ. , “Effect of Angiogenesis Inhibitors on Wound Healing in Patients with Ovarian Cancer: A Meta‐Analysis,” International Wound Journal 21, no. 3 (2024): e14737, 10.1111/iwj.14737.38468423 PMC10928245

The above article, published online on 11 March 2024, in Wiley Online Library (http://onlinelibrary.wiley.com/), has been retracted by agreement between the journal Editor in Chief, Professor Keith Harding; and John Wiley & Sons, Ltd. It came to the publisher's attention from a third party that a number of articles shared concerning similarities in format and structure. Following an investigation by the publisher, the retraction has been agreed on as the peer review and publishing process for this article were found to be manipulated. The authors disagree with the retraction.

Retraction: X. Li , D. Zeng , and J. Shi , “Effect of Angiogenesis Inhibitors on Wound Healing in Patients with Ovarian Cancer: A Meta‐Analysis,” International Wound Journal 21, no. 3 (2024): e14737, 10.1111/iwj.14737.38468423 PMC10928245

The above article, published online on 11 March 2024, in Wiley Online Library (http://onlinelibrary.wiley.com/), has been retracted by agreement between the journal Editor in Chief, Professor Keith Harding; and John Wiley & Sons, Ltd. It came to the publisher's attention from a third party that a number of articles shared concerning similarities in format and structure. Following an investigation by the publisher, the retraction has been agreed on as the peer review and publishing process for this article were found to be manipulated. The authors disagree with the retraction.

